# Implementation of a multicomponent intervention to prevent physical restraints in nursing home residents (IMPRINT): study protocol for a cluster-randomised controlled trial

**DOI:** 10.1186/s12877-015-0086-0

**Published:** 2015-07-21

**Authors:** Jens Abraham, Ralph Möhler, Adrienne Henkel, Ramona Kupfer, Andrea Icks, Charalabos-Markos Dintsios, Burkhard Haastert, Gabriele Meyer, Sascha Köpke

**Affiliations:** Martin Luther University Halle-Wittenberg, Medical Faculty, Institute of Health and Nursing Science, Magdeburger Str. 8, 06112 Halle (Saale), Germany; Witten/Herdecke University, Faculty of Health, School of Nursing Science, Stockumer Str. 12, 58453 Witten, Germany; University of Lübeck, Institute of Social Medicine, Nursing Research Unit, Ratzeburger Allee 160, 23538 Lübeck, Germany; University of Hamburg, MIN Faculty, Health Sciences, Martin-Luther-King-Platz 6, 20146 Hamburg, Germany; Heinrich-Heine-University Düsseldorf, Medical Faculty, Department of Public Health, Moorenstraße 5, 40225 Düsseldorf, Germany; mediStatistica, Lambertusweg 1b, 58809 Neuenrade, Germany

**Keywords:** Nursing homes, Physical restraints, Guidelines, Dementia

## Abstract

**Background:**

Physical restraints such as bedrails and belts are regularly applied in German nursing homes despite clear evidence showing their lack of effectiveness and safety. In a cluster-randomised controlled trial, the efficacy and safety of a guideline-based multicomponent intervention programme has been proven. The present study aims to evaluate the effectiveness of two different versions of the original intervention in nursing home residents in four different regions throughout Germany.

**Methods/Design:**

The study is a pragmatic cluster-randomised controlled trial comparing two intervention groups, i.e. (1) the updated original multicomponent intervention programme and (2) the concise version of the updated programme, with a control group receiving optimised usual care. The first intervention group receives an educational programme for all nurses, additional training and structured support for nominated key nurses, printed study material and other supportive material. In the second intervention group, nurses do not receive education as part of the intervention, but may be trained by nominated key nurses who have received a short train-the-trainer module. All other components are similar to the first intervention group. The control group receives the printed study material only. Overall, 120 nursing homes including approximately 10,800 residents will be recruited and randomly assigned to one of the three groups. The primary outcome is defined as the proportion of residents with at least one physical restraint after 12 months follow-up. The use of physical restraints will be assessed by direct observation. Secondary outcomes are the residents’ quality of life and safety parameters, e.g. falls and fall-related fractures. In addition, comprehensive process and economic evaluations will be performed.

**Conclusions:**

We expect a clinically relevant reduction in the proportion of residents with physical restraints. It is also expected that the process outcomes of this trial will enrich the knowledge about facilitators and barriers for the implementation of the multicomponent intervention programme.

**Trial registration:**

ClinicalTrials.gov: NCT02341898

## Background

Physical restraints (PR) such as bedrails and belts are commonly used in German nursing homes despite clear evidence showing their lack of effectiveness and safety [1–3]. A recent consensus statement defines PR as “any action or procedure that prevents a person’s free body movement to a position of choice and/or normal access to his/her body by the use of any method, attached or adjacent to a person’s body that he/she cannot control or remove easily” [4].

In Germany, legal regulations prohibit the use of PR with the exception of clearly delineated cases. In reality, PR remain common practice with a prevalence of about 25 % of residents with at least one PR. During a period of 12 months, approximately 40 % of residents are restrained at least once. Here, about one in ten residents receives a belt restraint and/or a fixed table to prevent standing up from a chair [5]. More important are the marked differences in the prevalence of PR between nursing homes, as shown in our earlier studies [1, 5]. These differences cannot be explained by the residents’ case mix or objectively measurable centre characteristics such as staffing level or staff training [5]. Most likely, the ‘philosophy’ or ‘culture’ of care (i.e. attitudes and beliefs of nursing staff) essentially determines the use of PR [5, 6]. In addition, the fact that there are nursing homes using very few or even no PR [5] indicate that adequate standard care in German nursing homes does not require the use of PR.

Nurses play a decisive role in the application of PR. Nurses claim to use PR for patient safety, especially for fall prevention [7–9] as well as to control challenging behaviour [5, 6]. However, the international evidence suggest that PR do not reduce falls and fall-related injuries, or control challenging behaviour [1–3, 7, 10] but may on the contrary increase the risk of falling by constraining mobility in this group of frail elderly persons. Furthermore, the use of PR is related to direct injuries through fatal entrapments as well as to reduced psychological well-being [7, 11–15]. Associations between PR and reduced quality of life as well as cognitive impairment have been documented [5, 16, 17]. Therefore, a ‘culture change’ has been demanded internationally for nursing homes, as the avoidance of PR is regarded mandatory from a professional point of view [18]. To ensure adequate nursing care and residents’ safety and quality of life, efforts to avoid PR in German nursing homes should be made a priority issue.

In our Cochrane review, we have summarised the current best evidence on programmes to reduce PR in nursing home residents [2, 19]. All the included studies assessed multicomponent interventions, including education for nurses, as core components. Results indicate that educational interventions might not be effective for reducing PR in nursing homes. In contrast, our previous cluster-randomised controlled trial of a guideline-based multicomponent intervention with 36 nursing homes resulted in a significant reduction of residents with PR after six months of follow-up [1]. This intervention is now ready for long-term implementation. However, the results of the intervention’s process evaluation indicated potential for improvement and modification of the intervention. For instance, it emerged that nursing home leaders essentially influence change processes of institutions as, for example, the attitudes of head nurses and the provision of specially trained key nurses seem to have contributed importantly to the success of the intervention [20]. Thus, a less extensive intervention might result in comparable or even more pronounced effects.

### Objectives

The main objective of the present study is to implement two versions of the guideline-based multicomponent intervention (updated original programme and concise version of the updated programme) and to investigate whether the interventions result in a reduction of PR use in nursing home residents when compared to optimised usual care. Furthermore, we intend to assess the impact of the interventions on residents’ quality of life and safety, i.e., falls and fall related fractures. A comparative health economic evaluation of the interventions will be conducted. Therefore, costs will be collected alongside the trial. Different process parameters, such as relatives' experiences, staff experiences, leaders' experiences and organizational culture will be assessed. We also aim to evaluate factors impeding or facilitating implementation of interventions.

## Methods/Design

### Study design

**Fig. 1:**
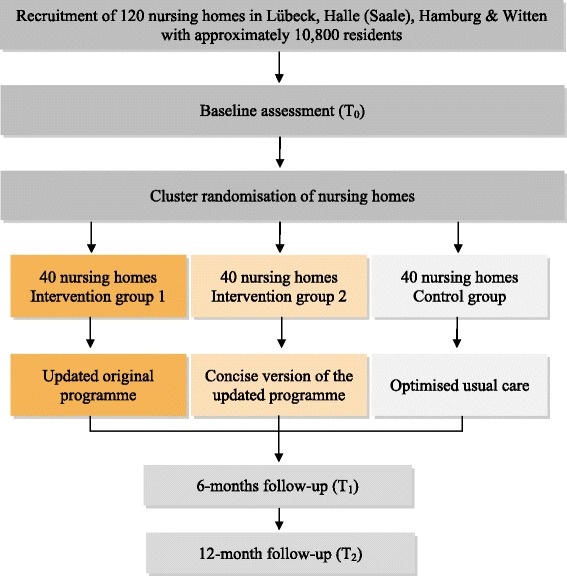
Flowchart for the cluster-randomised controlled trial

The IMPRINT (Implementation of a Multicomponent intervention to Prevent Physical Restraints In Nursing home residenTs) study is a pragmatic, cluster-randomised controlled trial with three parallel groups and a 12-months’ follow-up. A total of 120 nursing homes will be randomised equally either to one of the two intervention groups, i.e. (1) to the updated original multicomponent intervention programme or (2) to the concise version of the updated programme, or to the control group receiving optimised usual care (see Fig. 1).

### Preparatory work

In a first step, the evidence-based guideline [21] was updated from April to October 2014. Methodological steps are comparable to those taken in the primary study [22]. Overall, five online consensus meetings with a multidisciplinary guideline development group were held and 22 statements approved on relevant interventions to avoid PR use. In addition, all of the supportive materials were updated. The updated guideline and material were reviewed by three external peer reviewers familiar with guideline development and physical restraints. The concise version of the updated programme was developed, based on the in-depth analysis of the initial process evaluation [1, 20].

All of the study material was pilot-tested in three focus groups with legal guardians and/or relatives (*n* = 18), nursing staff (*n* = 12), nursing home managers (*n* = 8), relatives and nursing home residents (*n* = 3), one psychiatrist and one judge. The concise version of the intervention and also the instruments for data collection were pilot-tested in two nursing homes in Schleswig-Holstein in order to test the feasibility of the study procedures and material.

### Participants and recruitment

#### Inclusion and exclusion criteria

##### Cluster level

Clusters are defined as nursing homes or units working independently within large nursing homes. No specific inclusion and exclusion criteria will be applied for clusters.

##### Resident level

All of the residents living in the nursing homes and present on the day of data collection will be included. All of those newly admitted to clusters during follow-up and present on the day of follow-up data collection will also be included in the study. Newly admitted residents not present on the day of PR assessment will not be included.

##### Recruitment of study centres and study participants

Overall, 120 clusters with an expected mean of 90 residents will be recruited in the catchment areas of Lübeck (Northern Germany), Halle (Saale) (Eastern Germany), Witten (Western Germany) and Hamburg (Northern Germany). Approx. 30 clusters per area will be included. The facilities will be contacted in a random order selected for each study region from online nursing home registers. The nursing homes will be invited to take part in the study via postal mail and a subsequent telephone call. In addition, the study will be presented personally to the nursing home managers.

### Interventions

#### Updated original programme

The intervention (1) is an updated version of the original guideline-based multicomponent programme which has been evaluated successfully in an earlier cluster-randomised controlled trial [1]. The intervention consists of a single information session for all nurses (60 to 90 min) including information on PR with regard to background, risks and alternatives. Intensive training (one-day training workshop and half-day counselling) followed by structured support for nominated key nurses will also be carried out. Furthermore, printed study material (short version of the guideline; information brochures for nurses, legal guardians, general practitioners, relatives and others) and supportive material i.e. poster, mugs and pencils will be provided.

#### Concise updated programme

For the concise intervention, education for nurses is only optional and will be carried out by the nominated key nurses who will receive additional train-the-trainer education. Apart from that, the concise updated programme is equivalent to the updated original intervention.

#### Control group

The control group will receive optimised usual care. The nursing homes in the control group will be provided with printed study material only. Apart from the experimental interventions, control group and intervention group clusters will be treated equally. The intervention components for each group are displayed in Table 1.

**Table 1:** Components of the study interventions

Components of the interventions	Arms		
	Intervention group 1 (updated original programme)	Intervention group 2 (concise version)	Control group
Educational programme for all nurses	✓	-	-
Training and structured support for nominated key nurses	✓	✓	-
Facultative train-the-trainer module for key nurses	-	✓	-
Printed study material	✓	✓	✓
Supportive material (poster, mugs and pencils)	✓	✓	-

### Randomisation

Interventions will be implemented at the institutional level. Therefore, randomisation will be performed on a cluster level. Clusters will be allocated 1:1:1 between intervention and control arms. For the random assignment, computer-generated lists will be used with blocks of six, nine, and twelve nursing homes. The randomisation list will be generated by an independent external biometrician (BH). Because regional differences may influence the outcomes, randomisation will be stratified by region (Lübeck, Halle (Saale), Hamburg, and Witten). Allocation concealment will be ensured through assignment by an external independent researcher, who will inform the clusters about group allocation.

### Outcome measures

The primary outcome is the proportion of residents with at least one PR after twelve months. Data will be collected through direct observation at three measurement points: before randomisation (T_0_), after six months (T_1_) and after twelve months (T_2_). Observations at T_1_ and T_2_ will be performed by blinded research assistants. In contrast to our earlier study [1], observations will be performed only twice per day (morning and evening) instead of three times per day for reasons of feasibility.

Secondary outcomes are the residents’ quality of life and numbers of falls and fall-related fractures. The residents’ quality of life will be assessed using the German version [23] of the Quality of Life-Alzheimer’s Disease – QoL-AD [24, 25] at T_0_ and T_2_. This valid instrument has recently demonstrated its applicability and practicability in more than 2,000 people with dementia assessed in a European study [26]. For reasons of feasibility, a randomly chosen subgroup of 10 % of the residents will be selected. Proxy ratings will be done by nursing staff. Falls and fall-related fractures will be assessed by prospective documentation. In Germany, nursing homes are obliged to document this information.

Supported by the nurses, baseline data will be collected before randomisation for all residents living in the clusters. Here, characteristics of nursing homes and residents will be assessed including nurses’ proxy rating of residents’ cognitive status and challenging behaviour. The Dementia Screening Scale – DSS [27] will be used for assessing residents’ cognitive status. Challenging behaviour will be assessed using a modified German version of the Cohen-Mansfield Agitation Inventory – CMAI [28–30] which was already applied in previous studies [1, 5]. An abbreviated baseline assessment will be performed for residents admitted during the study period and present at the day of the follow-up data collection.

### Process evaluation

For generalizability of the study results and to support future implementation of the intervention, a comprehensive assessment of process measures is indispensable [31, 32]. Therefore, different process parameters will be assessed on cluster and individual levels respectively (see Table 2). The recruitment procedure will be documented on cluster level, including documentation of the information provided and the reasons for participation or non-participation. The documentation will include the results on the comprehensibility of the information material assessed during the pre-tests and also the results of the piloting phases. Contextual aspects will be recorded with respect to crucial structure- and process-related features and modifications at all three measurement points. Organizational culture will be evaluated at baseline and after twelve months in a random sample of 10 % of nurses and one leader per cluster using the German version of the “Organizational Culture Assessment Instrument” derived from the “Competing Values Framework” [33]. In a subgroup of 10 % randomly chosen nurses per cluster, knowledge and self-efficacy concerning PR will be assessed at baseline and after six and twelve months, using a self-developed questionnaire based on a previously applied questionnaire addressing nurses’ self-efficacy [1]. Legal guardians´ attitudes towards PR will be assessed at baseline and after twelve months on the legal guardians in a subgroup of 10 % of randomly chosen residents within each cluster, using the “Maastricht Attitude Questionnaire” [34, 35]. Intervention fidelity will be determined by structured documentation for each educational session and for each study nurse`s counselling session with the key nurses. All participants in the educational sessions will be asked to fill in the above-mentioned questionnaire assessing self-efficacy, knowledge and satisfaction. Furthermore, key nurses will be asked to document the crucial factors related to the intervention in a diary. Use of and demand for study material will be documented after six and twelve months. After twelve months, awareness of the intervention will be assessed by a short survey in a subgroup of three randomly selected nurses per cluster. Attitudes and experiences related to the maintenance of the intervention, including perceived barriers and facilitators, will be collected through focus group interviews with leaders/key nurses, nurses, relatives, legal guardians and residents. Additionally, structured final interviews on experiences, additional strains and unintended consequences will be conducted with key nurses and one leader per cluster after twelve months. The confidence of the residents in relation to the reduction of PR will be addressed in an additional multiple case study.

**Table 2:** Process evaluation

Focus	Documentation/Assessment	Measurement point
Comprehensibility/usability of information material	3 Focus groups: nurses, legal guardians, relatives	pre-test, prior to t_0_
Feasibility of the intervention	Piloting in 2 nursing homes:	Piloting, prior to t_0_
	1/intervention arm	t_0_
Recruitment procedure	Protocol/region	t_0_
Information of clusters on study sequence	Protocol/cluster	t_0_
	Information material: flyers, leaflets for leaders, nurses, residents	t_0_
Reasons for non-participation or drop-out	Structured inquiry and documentation of reasons	t_0_-t_2_
Description of crucial structure- and process-related factors on cluster-level (e.g. size of the institution, nurse-to-resident-ratio, regulations for approaching to behavioral and psychological symptoms of dementia (BPSD), architectural features, motivation/reasons for participation)	CRF-Baseline data/cluster	t_0_
Social-demographic data, self-efficacy and knowledge about physical restraints	10 % nurses/cluster:	
	Questionnaire 1: Baseline-data nurses	t_0_
	Questionnaire 2: Self-efficacy	t_0_, t_1_, t_2_
	Questionnaire 3: Knowledge about physical restraints	t_0_, t_1_, t_2_
Organisational culture	1 leader + 10 % nurses/cluster:	
	Questionnaire 4: D-OCAI (German version of the “Organizational Culture Assessment Instrument” derived from the “Competing Values Framework”)	t_0_, t_2_
Conveyance of the intervention (intervention fidelity)	Trainer/educational session (all trainers, including key nurses):	t_0_ (immediately after the educational intervention)
	Structured protocol of each educational session	
Need for the intervention and application of training content	All Participants of the educational program:	
	Questionnaire → evaluation of the program	t_0_ (immediately after the educational intervention)
	-Self-efficacy	
	-Knowledge	
	-Satisfaction	
	Study Nurse:	
	Protocols of all key nurses´ contacts and conversations, in relation to the study nurse supervision; content of conversations (e.g. barriers/facilitators) + frequency/intensity of supervision	t_0_ first 3 months
	Key nurses:	
	Diary → To what extent was the intervention implemented?/How many training sessions were held?/How often did key nurses meet each other?	t_0_,-t_2_
	3 randomly selected nurses/cluster:	
	Short Survey → awareness of the intervention	t_2_
Structural changes/modifications on cluster level (e.g. architectural modifications; new regulations for approaching BPSD; introduction of specific strategies to reduce physical restraints; new equipment)	1 leader/cluster:	
	Short Survey → “structural modifications”	t_1_, t_2_
Staff fluctuation	1 leader/cluster:	
	Questionnaire → staff-fluctuation	t_1_, t_2_
Barriers and facilitators (How/To what extent was the intervention implemented? Changes in daily nursing routine? Attitude towards the intervention? Influence of key nurses and other nurses on the reduction of physical restraints? Use of/Demand for information material?)	Leaders/key nurses:	
	8 Focus groups; 1/region/intervention arm	t_2_
	Relatives, legal guardians, home advisory board:	
	8 Focus groups; 1/measurement point/region	t_1_, t_2_
	Key nurses + 1 leader/cluster:	
	Structured final interview (including focus on unintended consequences/adverse effects: Delay of procedures? Uncertainty/Fear? Additional strains?)	t_2_
Attitude of nurses towards the intervention (Changes in daily nursing routine? Delay of procedures? Changes of the working atmosphere/climate? Uncertainty/Fear? Additional strains?)	1 nurse/cluster:	
	Focus group interviews in all 3 intervention arms	t_2_
Attitude of legal guardians toward physical restraints	Subgroup of residents’ legal guardians/cluster:	
	Maastricht Attitude Questionnaire (MAQ)	t_0_, t_2_
Changes in residents’ behaviors	Proxy-assessment by nurses/all residents:	
	CRF – cognition (DSS), challenging behavior (CMAI)	t_0_, t_2_
	Proxy-assessment by nurses/10 % of residents/cluster:	
	Quality of Life (QoL-AD)	t_0_, t_2_
Use of/Demand for material (information material, incentives/image material)	Observation by members of the research team: How/To what extent are materials present and used?	t_1_, t_2_
	Documentation: Use of material/additional demand	t_1_, t_2_
Falls, fall-related fractures	CRF-documentation/resident	t_0_, t_1_, t_2_
Physical restraints (prevalence)	CRF-documentation of prevalence/resident	t_0_, t_1_, t_2_
Confidence of residents (How secure/insecure do residents feel in case of reduction of physical restraints?)	Additional Multiple Case Study	

### Health economic evaluation

The objective of the economic evaluation is to estimate the cost-effectiveness of the intervention in terms of additional costs per additional resident who is not being physically restrained. The economic evaluation will be performed from the perspective of the German social insurance (statutory health insurance and long-term care insurance). The incremental cost-effectiveness ratio (ICER) will be calculated, i.e. the ratio of the difference in costs between intervention and control groups divided by the difference in the proportion of residents without physical restraints. Effect parameters of the clinical trial will be used. During the study, cost parameters will be collected on intervention-related components as well as on outcome-related components. Cost parameters will be registered throughout the course of the study and during process evaluation. Resource use will be quantified, using a standardised protocol which is based on previously used protocols [1, 36]. Resource use and costs directly associated with the intervention will be derived from the study documentation. Costs explicitly associated with developing the information and training material, and conducting the study, such as the data collection, will not be taken into account.

### Sample size calculation

The primary analysis will consist of two baseline adjusted comparisons of PR prevalence after twelve months, each comparing one of both intervention groups with the control group.

A Bonferroni adjustment for testing two times will be applied, yielding a significance level of α = 0.05/2 = 0.025. The unit of observation in the primary analysis is the home (= cluster).

It is assumed that prevalence throughout the homes will be approximately normal distributed. The estimation of the required sample size is based on the *t*-test comparing the prevalence differences between two groups (intervention and control) from baseline to twelve months. The power of each test in the primary analysis is estimated separately under the same circumstances.

Based on data of the initial study [1], a sample size of 38 homes per group is needed to detect a difference in prevalence of 5 % after twelve months with 90 % power (β = 0.10) using a two-sided significance level of 2.5 % (α = 0.025) and assuming a standard deviation of σ = 6.0 in both groups. Furthermore, presuming a dropout rate of 5 %, a sample size of 40 clusters per group, i.e. a total of 120 clusters, with a mean cluster size of 90 residents is planned.

### Statistical analysis

Statistical analysis will follow the Good Clinical Practice (GCP) standards and will be conducted after the end of the follow-up by the blinded biostatistician (BH) in charge. Data analysis will be performed by intention to treat. In the primary analysis, the prevalence of restraints on both intervention groups will be compared to the control group on cluster level.

It is assumed that prevalence throughout the nursing homes will be approximately normal distributed.

The statistical tests will be adjusted for baseline prevalence by fitting a linear model with twelve-month prevalence of the clusters as the dependent variable and using baseline prevalence and two indicator variables of the intervention groups as the independent variables. Differences of prevalence changes between each intervention group and the control group will be estimated and statistically tested using contrasts of the model. Both tests will be performed using a two-sided level of significance of α = 2.5 %, which is adjusted for double testing by Bonferroni. Cluster adjustment will be ensured by using clusters as observation units. Based on the same linear model, a secondary analysis will estimate the contrast between the prevalence differences of both intervention groups. This result will be interpreted in a descriptive manner. A non-significant difference cannot be interpreted as the equivalence of both interventions. Possible equivalence tests do not reach enough power to get reasonable results.

Data will be described at all three measurement points (T_0_, T_1_, T_2_), stratified by treatment groups using frequency tables, means, standard deviations, percentiles and prevalence, depending on the distribution of the variables. Data on the residents will be described according to the resident level. Variances and confidence intervals of the outcomes of residents will be estimated cluster-adjusted using the intra-class correlation coefficient (ICCC) [37]. In a further secondary analysis, the time courses of the prevalence in the three groups will be investigated on resident level, adjusting for clusters and for repeated measurement. A generalized linear mixed model is fitted with binary dependent variable “application of PR”. Fixed effects are treatment group, time, interaction of treatment and time, and the baseline value of PR. Cluster is a random effect, in a secondary model the interaction cluster*time is also included as a further random effect. Adjustment for repeated measurement is performed by covariance patterns [38], primarily assuming a compound symmetry structure. Other covariance structures and possible exclusion of the interaction between treatment and time will be discussed comparing different models.

Secondary outcomes (quality of life on a 10 %-subpopulation; falls and fall-related fractures) will be described at each time documented, and stratified by treatment groups. Variances and confidence intervals are cluster-adjusted using ICCC [37]. Statistical comparisons between the treatment groups will be performed using (generalized) linear mixed models as described above for the primary outcome adjusting for the clusters and the repeated measurements.

Missing values on cluster level would only occur in the case of dropout homes, which, it is assumed, will be very rare and improbable. It is assumed that the documentation of PR at each investigation time will be nearly complete, so that there is no relevant selection bias from the estimated prevalence of PR of each home. Furthermore, in the secondary analyses the mixed models use all the available values of the residents and do not exclude residents because of partially incomplete values. It is not planned to impute missing values. The level of significance is 5 %, if not stated otherwise (e.g. in the primary analysis). All of the tests are performed two-sided.

Focus groups will be analysed by content analysis [39]. Two independent research team members will identify initial themes describing comparable aspects or meanings. To abstract the content, all initial themes will be labelled with codes. Based on the codes, categories and sub-categories will be developed. Coding and analysis will be discussed constantly. Disagreements about the themes will be resolved in a discussion. The content will be summarised and narratively described.

For the health economics analysis, mean costs as well as cost differences between intervention and control groups will be calculated. Cluster-adjustment will be conducted. Sampling uncertainty (95 % confidence intervals) will be estimated using bootstrap procedure because cost data are usually skewed. Effects will be taken from the documented events. ICER will be estimated in terms of costs per resident with avoided PR. The non-parametric bootstrap method will be employed to generate confidence intervals around the ICER estimate derived from the study sample [40, 41]. Uncertainty surrounding the ICER will also be presented on the cost-effectiveness plane and as the cost-effectiveness acceptability curve [42, 43]. Besides statistical uncertainty (sampling variation) with regard to costs and effects, every economic evaluation may contain some degree of data imprecision (e.g. resource costs/prices), which should be accounted for. To handle this type of uncertainty, a sensitivity analysis will be employed. In the sensitivity analysis, (uncertain) parameter(s) of the base-case analysis will be varied to determine whether changes in these parameters influence the results. We will report both, the revised point estimates and revised confidence intervals for costs, effectiveness, and cost- effectiveness that result from the sensitivity analyses.

### Data management

From earlier cluster-randomised controlled trials [1, 44, 45], various Standard Operating Procedures (SOPs) are available on the recruitment of nursing homes, Case Report Form (CRF) preparation according to GCP, data collection and data entry. SOPs for data audit and ethical issue management from a recent epidemiological study [26] will also be used. At the study centre in Lübeck, data will be entered electronically into a database by scanner-based software (TeleForm). During the scanning process and electronic recording, the data will be checked for plausibility. Incorrectly placed check marks or unclear text fields will be verified and corrected manually. For quality assurance, all digitally recorded study data will be checked individually and any changes or corrections will be documented. After freezing the data, any further changes to the database will be impossible. Data will be entered within one month after each measurement point.

Data audit will increase the credibility of the study and help to improve the data collection and archiving procedures. It will be carried out by trained researchers not engaged in the study, who will work according to a Data Audit Manual which follows GCP rules and was developed and proven in eight countries of a current European study [26].

Given the nature of the intervention, we do not expect serious adverse events. Thus, no interim analysis is planned and no stopping rules will be applied.

### Dissemination policy

We will publish the main study outcomes in an international, peer-reviewed journal and will present the results at scientific conferences. All results will be reported adhering to CONSORT Statement extension to cluster-randomised trials [46]. All trial information, e.g. procedures, material and results of the programme, will be freely available via the already existing trial website (http://www.leitlinie-fem.de). The homepage contains different freely accessible information material addressing researchers, clinicians, nursing staff, healthcare providers, and consumers. The programme will be offered to relevant healthcare providers throughout Germany. Furthermore, policy makers in Germany will be informed about the study by letter and an invitation to the presentation of the final results at one of the applicants’ universities.

### Ethical and legal considerations

The study protocol has been approved by the ethical committees of the Universities of Lübeck in January 2015 (no. 14–251) and Halle (Saale) in March 2015 (no. 2015–02). Written informed consent will be obtained from the managers of each participating nursing home before the start of the trial. Participating nursing homes may withdraw their consent at any time. As has been successfully carried out in previous trials [1, 5, 44, 45], no written informed consent from residents or their legal guardians will be required, as the investigators will have no direct access to the residents' data.

According to data protection regulations, a member of the nursing staff will accompany the investigators. Residents’ rooms will only be entered after the nurse has asked the resident if he or she agrees to be visited. Assessment of residents’ cognition, behaviour and quality of life will be performed by nurses’ proxy rating.

All resident-related data will be pseudonymised for the investigators by using a resident code number. Only the study coordinators at the nursing homes will have access to the names of the participating residents. All electronic data and CRFs will be archived securely for ten years at the University of Lübeck and will then be destroyed. The archive will not contain the residents’ names. Due to the results of our previous trial [1], we do not expect any risks for the participating residents. Good Clinical Practice will be fully guaranteed. Hence, a random sample of nursing homes will be visited and an external data auditor will personally check the data. Research assistants will be educated about correct data collection, data processing and data protection.

## Discussion

This cluster-randomised controlled trial will investigate the effectiveness of two versions of an already successfully evaluated guideline-based multicomponent intervention programme for the avoidance of PR in nursing homes. The first version is an update of the original guideline-based multicomponent programme and the second a corresponding concise version.

The study will include a large sample of 120 nursing homes with approximately 10,800 residents in four different regions throughout Germany. Therefore, for feasibility purposes some study procedures will be pragmatic, for instance proxy rating of residents’ quality of life in a randomly chosen subgroup. Apart from that, the rigorous study design with embedded mixed methods will ensure the validity and generalizability of the study results. Considering the kind of intervention, blinding of the nursing staff is not possible. However, the research assistants, who will collect PR data after randomisation, and the biostatistician will be blinded to group allocation.

We expect a clinically relevant reduction in the proportion of residents with PR as a result of the implementation of both interventions of the programmes. We also expect that the process outcomes of this trial will enrich our knowledge about promoting factors and barriers for implementation of this multicomponent intervention programme. If implementation is successful, the concise version of the updated programme will be a less extensive and potentially more cost-effective intervention for avoiding the use of PR and should be implemented in nursing homes throughout Germany. The comprehensive assessment of process measures and the health economics evaluation can facilitate future implementation of the intervention in routine care.

## Abbreviations

BPSD: Behavioral and psychological symptoms of dementia
CMAI: Cohen-mansfield agitation inventory
CRF: Case report form
D-OCAI: Organizational culture assessment instrument (German version)
DSS: Dementia screening scale
GCP: Good clinical practice
ICCC: Intra-class correlation coefficient
ICER: Incremental cost-effectiveness ratio
IMPRINT: Implementation of a multicomponent intervention to prevent physical restraints in nursing home residents
MAQ: Maastricht attitude questionnaire
PR: Physical restraints
QoL-AD: Quality of life in alzheimer’s disease
SOP: Standard operating procedure
